# Diagnostic performance of 3D cardiac magnetic resonance perfusion in elderly patients for the detection of coronary artery disease as compared to fractional flow reserve

**DOI:** 10.1007/s00330-022-09040-7

**Published:** 2022-08-19

**Authors:** Mihály Károlyi, Alexander Gotschy, Malgorzata Polacin, Sven Plein, Ingo Paetsch, Cosima Jahnke, Michael Frick, Rolf Gebker, Hatem Alkadhi, Sebastian Kozerke, Robert Manka

**Affiliations:** 1 Institute of Diagnostic and Interventional Radiology, University Hospital Zurich, Raemistrasse 100, 8091 Zurich, Switzerland; 2 Department of Cardiology, University Heart Center, University Hospital Zurich, Zurich, Switzerland; 3 Institute for Biomedical Engineering, University and ETH Zurich, Zurich, Switzerland; 4 Multidisciplinary Cardiovascular Research Centre & the Department of Biomedical Imaging Science, Leeds Institute of Cardiovascular and Metabolic Medicine, University of Leeds, Leeds, UK; 5 Department of Electrophysiology, HELIOS Heart Center Leipzig at University of Leipzig, Leipzig, Germany; 6 Department of Cardiology, Pneumology, Angiology and Intensive Care Medicine, University Hospital RWTH Aachen, Aachen, Germany; 7 German Heart Institute, Berlin, Germany

**Keywords:** Aged, Coronary angiography, Coronary artery disease, Fractional flow reserve, Myocardial perfusion imaging

## Abstract

**Objectives:**

In patients of advanced age, the feasibility of myocardial ischemia testing might be limited by age-related comorbidities and falling compliance abilities. Therefore, we aimed to test the accuracy of 3D cardiac magnetic resonance (CMR) stress perfusion in the elderly population as compared to reference standard fractional flow reserve (FFR).

**Methods:**

Fifty-six patients at age 75 years or older (mean age 79 ± 4 years, 35 male) underwent 3D CMR perfusion imaging and invasive coronary angiography with FFR in 5 centers using the same study protocol. The diagnostic accuracy of CMR was compared to a control group of 360 patients aged below 75 years (mean age 61 ± 9 years, 262 male). The percentage of myocardial ischemic burden (MIB) relative to myocardial scar burden was further analyzed using semi-automated software.

**Results:**

Sensitivity, specificity, and positive and negative predictive values of 3D perfusion CMR deemed similar for both age groups in the detection of hemodynamically relevant (FFR < 0.8) stenosis (≥ 75 years: 86%, 83%, 92%, and 75%; < 75 years: 87%, 80%, 82%, and 85%; *p* > 0.05 all). While MIB was larger in the elderly patients (15% ± 17% vs. 9% ± 13%), the diagnostic accuracy of 3D CMR perfusion was high in both elderly and non-elderly populations to predict pathological FFR (AUC: 0.906 and 0.866).

**Conclusions:**

3D CMR perfusion has excellent diagnostic accuracy for the detection of hemodynamically relevant coronary stenosis, independent of patient age.

**Key Points:**

*• The increasing prevalence of coronary artery disease in elderly populations is accompanied with a larger ischemic burden of the myocardium as compared to younger individuals.*

*• 3D cardiac magnetic resonance perfusion imaging predicts pathological fractional flow reserve in elderly patients aged ≥ 75 years with high diagnostic accuracy.*

*• Ischemia testing with 3D CMR perfusion imaging has similarly high accuracy in the elderly as in younger patients and it might be particularly useful when other non-invasive techniques are limited by aging-related comorbidities and falling compliance abilities.*

**Supplementary Information:**

The online version contains supplementary material available at 10.1007/s00330-022-09040-7.

## Introduction

The worldwide population aged ≥ 65 years is projected to nearly double within the next three decades, while the number of people aged 80 years and older is expected to triple until 2050 (“World Population Ageing 2020 Highlights” by the United Nations Department of Economic and Social Affairs, published online in 2020). The prevalence of coronary artery disease (CAD) also increases with age and the pre-test probability of CAD is highest among individuals older than 70 years with angina pectoris or angina equivalent symptoms [1]. Therefore, it is essential to have widely available and reliable diagnostic tests suitable for this population. At the same time, due to age-related limited compliance and increased prevalence of other comorbidities, patients with advanced age are often underrepresented in large-scale clinical trials and until now a very limited number of studies have addressed the performance of pharmacological stress tests in this population [2, 3].

Cardiac magnetic resonance (CMR) myocardial perfusion imaging allows for ruling out myocardial ischemia with a high degree of accuracy and is supported by the latest guideline of the European Society of Cardiology (ESC) for this indication [4]. Conventional CMR myocardial perfusion uses 2D acquisition of 3 short-axis slices of the left ventricle (LV). Concurrently, in the case of relevant coronary artery disease, the decision on revascularization has to take into consideration not only the anatomical localization but also the amount of ischemia [5]. Therefore the use of a 3D technique with full LV coverage might be beneficial in the elderly population. Recent technical advances allow for 3D perfusion CMR, which overcomes this limitation and would make it the ideal test for myocardial ischemia in the elderly population. Importantly, the accuracy of 3D CMR stress perfusion has been recently validated in large-scale multi-center trials [6–8]. Yet, the performance of perfusion CMR, particularly with the 3D technique in elderly patients, has not been assessed yet.

Given this background, we investigated the performance of 3D cardiac magnetic resonance perfusion in patients aged ≥ 75 years for the detection of coronary artery disease as compared to the current reference standard invasive coronary angiography using fractional flow reserve (FFR). We hypothesized that 3D cardiac magnetic resonance perfusion is as accurate in elderly patients as in younger individuals.

## Materials and methods

### Study population

All patients included in the current study were retrospectively enrolled from a research database of a previously published multi-center trial, who underwent 3D perfusion CMR study and invasive coronary angiography with fractional flow reserve (FFR) due to known or suspected CAD [6–9]. Imaging studies were performed between 2009 and 2013 in five European centers. Known contraindications for perfusion CMR, such as conflicting metallic foreign body, claustrophobia, bronchial asthma, or high-degree AV-block, were considered contraindications for the original study. No adverse reactions from performing the perfusion CMR study or invasive FFR were documented. For the current study, elderly patients were selected, defined as patients with the advanced age of 75 years or older [10]. An additional subgroup analysis was performed for octogenarians, aged ≥ 80 years. Findings were compared with the results of younger individuals < 75 years old, who underwent the same study protocol within the inclusion timeframe. All 416 patients in this study were included in the previously published trial [9]; nevertheless, the diagnostic utility of 3D perfusion CMR in the sub-group of elderly patients has not been analyzed before and a completely renewed analysis of data was performed for this purpose. The study protocol was approved by local ethics committees in each participating center and written informed consent from patients was obtained.

### Myocardial perfusion cardiac magnetic resonance imaging

Subject to availability at the participating sites, 1.5-T or 3.0-T magnetic resonance scanner systems were used from a single vendor (Philips). Cardiac synchronization was provided using vector ECG and signal reception was ensured using either 5-, 6-, 28-, or 32-channel torso coil arrays. Cine images at standard axes and whole-heart 3D first-pass perfusion at stress and rest in short-axis (SA) orientation covering the complete left ventricle from base to apex were acquired. A saturation-recovery gradient-echo pulse sequence was used for the perfusion study with the following parameters: 350 × 350 mm FOV, 2.3 × 2.3 × 10.0 mm^3^ voxel size reconstructed to 2.0 × 2.0 × 5.0 mm^3^, TR/TE 1.9/0.8 ms, flip angle of 15^o^, saturation delay of 150 ms and partial Fourier acquisition. A volume of 16 slices was acquired pro heartbeat using 10 × undersampling resulting in a net acceleration of 7 ×. The acceleration technique used for 3D cardiac perfusion imaging was published previously [11]. Finally, late gadolinium enhancement imaging (LGE) 15 min after the same SA orientation was performed, while a Look-Locker sequence was applied to define the ideal inversion recovery pre-pulse delay for LGE imaging. Participants were instructed to restrain caffeine intake 24 h prior to the exam. The standard dose (140 μg/kg/min) of adenosine was administered intravenously ≥ 3 min for pharmacological stress and 0.1 mmol/kg of a gadolinium-based contrast agent followed by a bolus saline injection was used for data acquisition. Using the same data acquisition planning, stress as well as rest perfusion study was acquired in inspiration breath-hold with allowed shallow expiration, supposing that breath-hold can not be sustained during the complete data acquisition.

### Assessment of myocardial ischemia with perfusion CMR

Analysis of CMR perfusion exams was performed as part of the original multi-center studies in a core laboratory using dedicated software (Extended WorkSpace, Philips Healthcare). Readers were blinded to both clinical and angiographic data. The number of acquired SA slices during the perfusion study was adapted for the individuals’ heart size (ranging between 9 and 16 slices). Thereafter, acquired slices with > 75% circumferential LV myocardium and clear delineated LV cavity during first-pass perfusion imaging were further analyzed, while each slice was divided into 6 segments. Segmental myocardial ischemia was assessed visually, defined as a perfusion deficit during first-pass stress perfusion with at least 25% transmurality and persistent for ≥ 3 consecutive dynamics, without corresponding defects on rest perfusion or enhancement on LGE. Moreover, the transmurality of each myocardial segment positive for ischemia was further grated using a 4-point scale (1: 25%; 2: 26–50%; 3: 51–75%; 4: 76–100%) and converted into a perfusion score, which was normalized to the heart size with an average of 12 myocardial slices in correspondence with the 17-segment model of the American Heart Association [12].

Myocardial ischemic burden (MIB), defined as the percentage of hypoenhanced myocardium relative to total myocardial volume, was further quantified using a semi-automated method (GTvolume, Gyrotools). For this purpose, LV endo- and epicardial borders were contoured manually on the dynamic frame of stress perfusion study with the maximal myocardial hypoenhancement extent throughout peak signal intensity of remote myocardium and ischemic myocardial area was quantified with a threshold-based method using > 2 standard deviation beneath remote myocardium signal intensity, expressed as ischemic burden (IB). A disk summation method was used to calculate total myocardial volumes. Myocardial scar burden (SB) defined as hyperintense tissue on the LGE scans in comparison to remote myocardium was quantified similarly using the same software, and in case of overlap, the myocardial scar was subtracted from the myocardial hypoattenuation and was normalized to LV volume. MIB equals IB – SB.

### Analysis of myocardial ischemia with invasive QCA and FFR

X-ray coronary angiography was performed according to standard techniques and routine protocol at each site. At least two orthogonal views of every major coronary artery and their side branches were obtained. Quantitative coronary angiography (QCA) was assessed offline (Philips Inturis CardioView, QCA V3.3, Pie Medical Imaging) in a core laboratory blinded to CMR and clinical data. Significant stenosis was defined as a luminal narrowing ≥ 50% of the coronary artery. FFR was assessed with a 0.014-in. coronary pressure sensor-tip wire in all vessels present with diameter stenosis between ≥ 50 and ≤ 80% in two orthogonal views and a luminal diameter of at least 2 mm. A functional relevant stenosis was defined as exhibiting an FFR value of < 0.8. A luminal stenosis > 80% or total occlusion was considered hemodynamically relevant and no FFR was performed. No routine-controlled pull-back procedure was performed.

### Statistical analysis

The Shapiro-Wilk-test was used to assess the normality of continuous variables. Continuous variables are reported as mean ± standard deviation. Categorical variables are expressed as numbers and percentages. Student’s t-test was used for comparison of normally distributed continuous variables between stress and rest. Non-normally distributed continuous data was compared using the Mann-Whitney U test. Sensitivity, specificity, positive predictive value, and negative predictive value were calculated on a patient level with correspondent 95% confidence intervals (95% CI). Chi-squared test was used to compare categorical data. All tests were two-tailed and a *p* value < 0.05 was considered statistically significant. Diagnostic accuracy was determined with receiver–operator–characteristics (ROC) curve analysis (area under the curve, AUC). Statistical analysis was performed using SPSS software (SPSS version 23).

## Results

### Patient demographics

The study cohort consisted of 56 patients at the age of 75 years or older (mean age 79 ± 4 years, 35 male) and 360 patients aged below 75 years (mean age 61 ± 9 years, 262 male). Patient characteristics and clinical data are summarized in Table 1.

**Table 1:** Characteristics and clinical data of study population aged < 75 years and ≥ 75 years

	< 75 years	≥ 75 years	*p* value
	*n* = 360	*n* = 56	
Baseline characteristics			
Age, mean ± SD	61 ± 9	79 ± 4	
Male, *n* (%)	262 (73)	35 (63)	0.113
BMI (kg/m^2^), mean ± SD	28 ± 4	26 ± 4	0.057
Cardiovascular risk factors			
Hypertension, *n* (%)	269 (75)	49 (88)	0.036
Diabetes, *n* (%)	79 (22)	11 (20)	0.697
Dyslipidemia, *n* (%)	240 (67)	33 (59)	0.332
Smoker, *n* (%)	141 (39)	15 (27)	0.075
Family risk of CAD, *n* (%)	105 (29)	8 (14)	0.020
Regular medication			
ACE-inhibitor, *n* (%)	191 (53)	33 (59)	0.412
ARB, n (%)	68 (19)	11 (20)	0.903
Beta-blocker, *n* (%)	241 (70)	46 (82)	0.022
Calcium-antagonist, *n* (%)	66 (18)	19 (34)	0.007
Diuretic, *n* (%)	47 (13)	18 (32)	0.001
Nitrate, *n* (%)	24 (7)	7 (13)	0.210
Statin, *n* (%)	263 (73)	43 (77)	0.556
CMR characteristics			
LVEF (%), mean ± SD	58 ± 9	58 ± 7	0.520
LV end-diastolic volume (mL), mean ± SD	142 ± 47	128 ± 36	0.028
LV end-systolic volume (mL), mean ± SD	63 ± 36	56 ± 23	0.158
End-diastolic septum thickness (mm), mean ± SD	11 ± 2	11 ± 3	0.321
End-diastolic lateral wall thickness (mm), mean ± SD	10 ± 1	10 ± 1	0.464
CMR perfusion parameters			
Stress			
Heart rate (bpm), mean ± SD	84 ± 15	79 ± 12	0.021
Systolic blood pressure (mmHg), mean ± SD	128 ± 21	134 ± 21	0.065
Diastolic blood pressure (mmHg), mean ± SD	71 ± 10	69 ± 9	0.161
Rest			
Heart rate (bpm), mean ± SD	67 ± 11	67 ± 11	0.833
Systolic blood pressure (mmHg), mean ± SD	129 ± 19	136 ± 19	0.008
Diastolic blood pressure (mmHg), mean ± SD	73 ± 10	71± 11	0.265

*BMI*, body mass index; *CAD*, coronary artery disease; *ACE*, angiotensin-converting; *ARB*, angiotensin receptor blocker; *LV*, left ventricle; *LVEF*, left ventricle ejection fraction; *SD*, standard deviation.

### Diagnostic accuracy of CMR stress perfusion

The prevalence of relevant coronary artery stenosis, defined as ≥ 50% luminal narrowing, was 70% (39/56 patients) among the elderly and 54% (195/360 patients) in the group aged below 75 years. Hemodynamically relevant stenosis, specified with FFR using 0.8 as a cut-off value, was 68% (38/56 patients) and 52% (186/360 patients) in the population aged 75 years or older and in the population aged below, respectively.

**Fig. 1:**
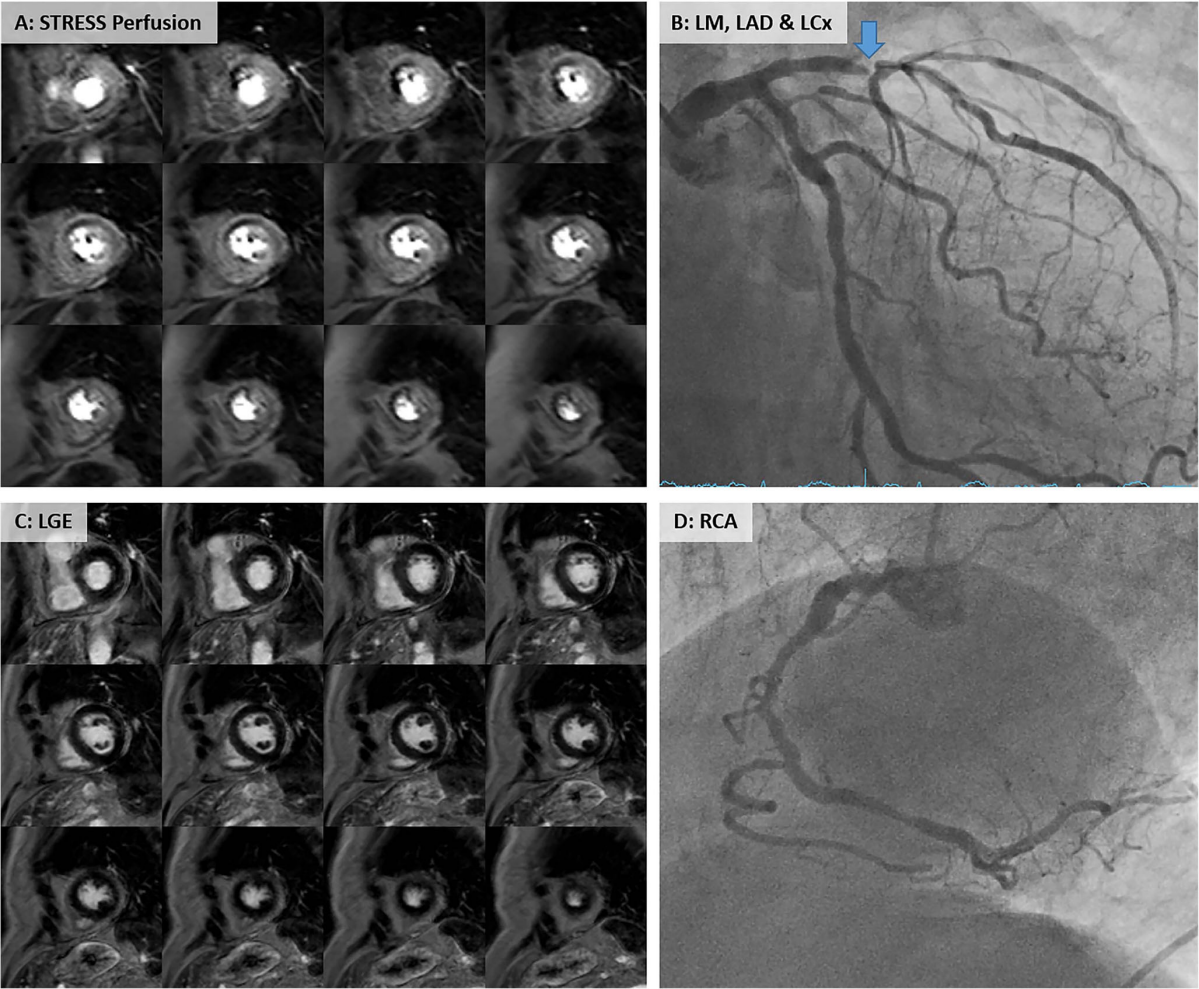
Example of 3D stress perfusion with CMR in an elderly patient. 3D stress perfusion of an 85-year-old female patient (**A**) shows a large perfusion defect in the anterior and antero-septal segments caused by a subtotal occlusion of the proximal LAD (blue arrow) as shown in the coronary angiogram (**B**). Late gadolinium enhancement (LGE) images confirm preserved viability of the whole left ventricular myocardium (**C**). In agreements with the perfusion and LGE results, there is no significant stenosis of the RCA (**D**). LM, left main; LAD, left anterior descending; LCx, left circumflex; RCA, right coronary artery

Among the individuals aged ≥ 75 years, ischemia testing with 3D CMR perfusion was positive in 68% (38/56 patients) as compared with FFR. The correspondent sensitivity and specificity of dynamic 3D CMR to detect ischemia using FFR as a reference were 87% (95% CI: 72–96) and 83% (95% CI: 59–96) in the population aged ≥ 75 years and 87% (82–92) and 80% (73–86) among patients aged < 75 years, respectively. There was no significant difference in the diagnostic performance of 3D CMR perfusion between the different age groups, as summarized in Table 2*.* No significant differences were seen in octogenarians (≥ 80 years) compared with patients < 80 years of age (see Supplemental material). Examples of stress perfusion imaging and corresponding invasive angiography are shown in Fig. 1.

**Table 2:** Summary of diagnostic performance of 3D stress perfusion with CMR

	< 75 years	≥ 75 years	*p* value
	*n* = 360	*n* = 56	
FFR			
Sensitivity, % (95% CI)	87 (82–92)	87 (72–96)	0.893
Specificity, % (95% CI)	80 (73–86)	83 (59–96)	0.727
Positive predictive value, % (95% CI)	82 (78–86)	92 (80–97)	0.162
Negative predictive value, % (95% CI)	85 (80–89)	75 (56–87)	0.206

*FFR*, fractional flow reserve; *CI*, confidence interval

### Quantitative analysis of CMR stress perfusion

**Fig. 2:**
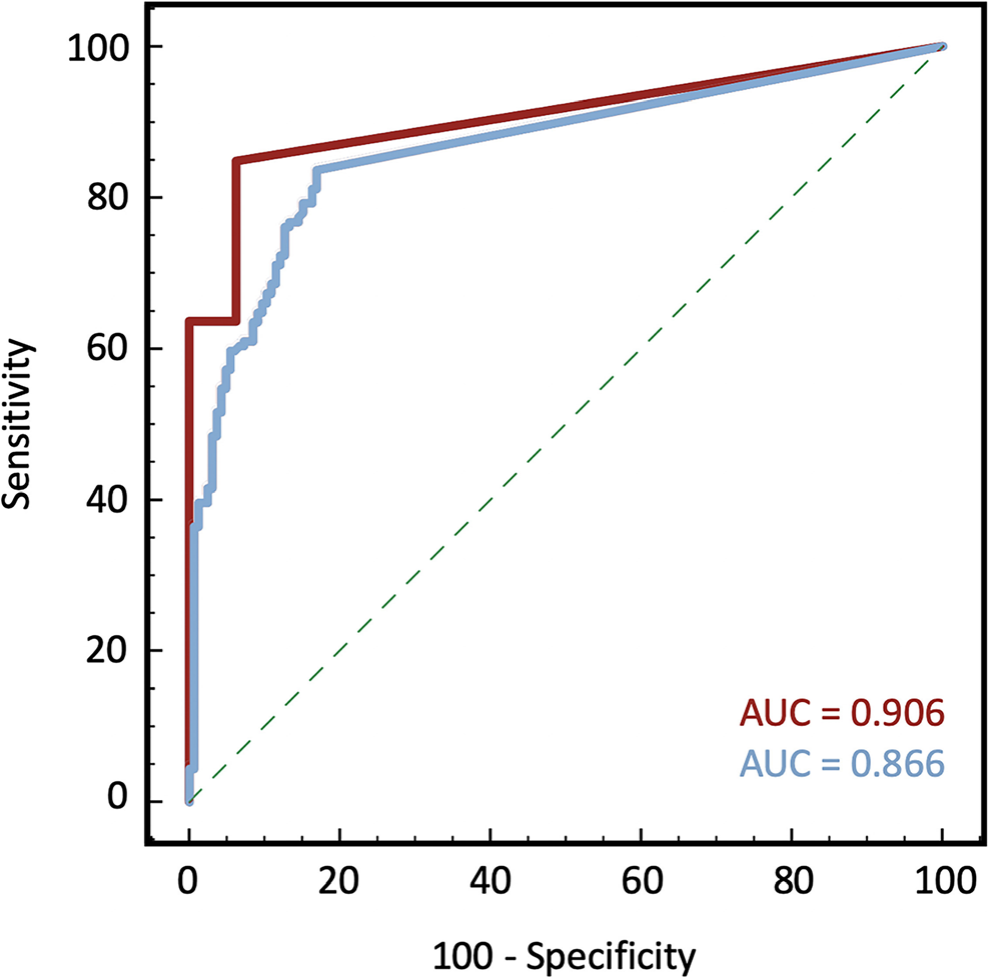
Diagnostic ability of quantitative 3D stress perfusion CMR as compared to gold-standard FFR. Receiver operating characteristic (ROC) curves of predicting pathological FFR from 3D stress perfusion CMR in the populations aged ≥ 75 years (red line) and < 75 years (blue line), quantified as myocardial ischemic burden and given as a percentage relative to whole myocardial volume. *AUC*, area under the curve

MIB was larger among patients ≥ 75 years old than patients aged < 75 years, although the difference did not reach the level of statistical significance (15% ± 17% and 9% ± 13%, respectively, *p* = 0.055). The AUC to predict pathological FFR for MIB in the elderly population was 0.906 (95% CI: 0.822-0.991) versus 0.866 (95% CI: 0.824-0.907) in those < 75 years. Diagnostic findings are summarized in Table 3. ROC curves of the two subgroups are displayed in Fig. 2 and those for octogenarians are in the Supplemental material.

**Table 3:** Diagnostic findings

	< 75 years	≥ 75 years	*p* value
	*n* = 360	*n* = 56	
CMR findings			
Ischemia, *n* (%)	198 (55)	36 (64)	0.193
Ischemic burden (%), mean ± SD	11 ± 14	18 ± 18	0.026
Scar, *n* (%)	51 (14)	3 (5)	0.068
Scar burden (%), mean ± SD	1.5 ± 6	0.7 ± 2	0.877
MIB (%), mean ± SD	9 ± 13	15 ± 17	0.055
Invasive coronarography findings			
CAD (> 50% luminal stenosis), *n* (%)	195 (54)	39 (70)	0.030
Single-vessel disease, *n* (%)	104 (29)	23 (41)	0.066
Multi-vessel disease, *n* (%)	92 (26)	16 (29)	0.632
Pathological FFR (< 0.8), *n* (%)	186 (52)	38 (68)	0.024

*MIB*, myocardial ischemic burden = ischemic burden - scar burden; *CAD*, coronary artery disease; *FFR*, fractional flow reserve; *SD*, standard deviation

## Discussion

Our study focused on the role of myocardial ischemia testing in the elderly population (aged ≥ 75 years) using 3D CMR perfusion. We compared our findings with the reference standard invasive coronary angiography using FFR. 3D CMR perfusion showed excellent diagnostic accuracy for the detection of hemodynamically significant lesions (FFR value < 0.8), independent of patient age. Furthermore, we demonstrated that quantitative ischemia analysis with 3D CMR perfusion is a precise technique for the prediction of hemodynamically relevant coronary artery lesions, as compared with FFR. Importantly, our findings remained consistent in the subgroup analysis of octogenarian patients (aged ≥ 80 years).

Recently, a substantial decrease (from 45 to 15%) in the prevalence of obstructive CAD was recognized among patients with angina-like symptoms [1], which is well represented in the new pre-test-probability (PTP) categories of the latest (2019) guidelines of the European Society of Cardiology (ESC) for the diagnosis and management of chronic coronary syndromes [4]. Hence, a shift of non-invasive CAD testing towards patients with higher pre-test probabilities is further supported, which has particular implications for individuals at advanced age. For example, based on the earlier ESC guideline, the diagnosis of stabile CAD could be established in a ≥ 70-year-old male patient presenting with typical chest pain considering only the high (> 85%) PTP [13], while the newly reduced (52%) PTP of this patient supports further non-invasive testing for establishing the diagnosis of CAD [4]. Moreover, besides women with non-anginal symptoms or dyspnea, all ≥ 70-year-old symptomatic individuals now fall into the categories (PTP > 15%), where non-invasive testing is most likely beneficial [4]. Elderly people often have reduced exercise capabilities; therefore, anatomical or pharmacological stress tests are preferred. The choice of a non-invasive test should be established based on the PTP of CAD and certain patient characteristics. The prevalence of coronary artery calcium significantly increases with aging, which substantially reduces the specificity of coronary CT angiography in this population [14]. Moreover, the incidence of arrhythmias limiting the diagnostic image acquisition with coronary CTA increases with age, whereas up to 10% of octogenarians is expected to develop atrial fibrillation [15]. Contrary to coronary CTA, the diagnostic performance of pharmacological stress tests, such as adenosine stress CMR is little affected by cardiac arrhythmias [16]. Limited compliance and other comorbidities, such as impaired renal function or inability to breath-hold may limit equally anatomical and functional tests in this population. Importantly, the rule-in power of functional ischemia tests is stronger than that of an anatomical test, which is especially relevant among patients with a known or higher risk of obstructive CAD. Accordingly, besides positron emission tomography, CMR can rule in hemodynamically significant coronary lesions in high-risk patients with a high degree of accuracy [1]. CMR may also exclude alternative causes for symptoms and provides valuable information about biventricular size and function as well as a scar, which are related to prognosis and therefore should be part of the diagnostic work-up [4]. These properties make CMR an ideal test for elderly patients. In addition, as LV remodeling advances with age, full LV coverage with 3D stress perfusion may have an additional benefit in older individuals over conventional 2D techniques.

There is a paucity of data specifically evaluating CMR stress perfusion in elderly patients aged 75 years or above. An early study performed on 309 individuals ≥ 70 years confirmed the safe applicability of dobutamine and adenosine stress CMR; however, the diagnostic accuracy of stress perfusion with CMR was not established [17]. Patients included in previous large-scale clinical trials using stress CMR were considerably younger, such as in the CE-MARC 1 and 2 with a mean age of 60.2 ± 9.7 and 56.3 ± 9.0 years [18, 19] and in the MR-IMPACT I and II trials of 60.7 ± 10.2 and 60.0 ± 10.3 years [20, 21], respectively.

In the present study, we demonstrated that the high sensitivity and specificity values of 3D stress perfusion CMR to detect ischemia remain consistent in the elderly population (87% and 83%), as compared to younger individuals (87% and 80%). Our results are comparable with a recent meta-analysis where the pooled sensitivity and specificity values of perfusion stress CMR to identify functionally significant CAD were 89% (95%CI: 85–92%), 87% (95%CI: 83–91%), respectively, whereas the mean age of the included cohort was 63 ± 3 years [22].

Notably, we observed a trend towards a higher positive predictive value (PPV) of 3D stress CMR in individuals 75 years and older, than in younger patients, as compared to FFR, which however was not statistically significant (92% vs. 82%, *p = *0.162). The markedly high PPV might however justify the benefit of this ischemic test in the elderly population, where the prevalence of 50–90% luminal coronary stenosis with potential therapeutic implications is most prevalent. This finding was further supported by our quantitative analysis, where a larger MIB was found in the older population, although this result did not reach statistical significance (15 ± 17% vs. 9 ± 13%, *p* = 0.055). Our findings need to be validated in larger clinical trials. Importantly, ≥ 10% reversible myocardial ischemia of the entire LV myocardium is associated with an elevated (> 3%) annual risk for myocardial infarction, or cardiovascular death, as compared with a normal test (≤ 1%) [23, 24], which supports the benefit of added ischemia quantification with 3D stress perfusion CMR [25]. Importantly, MIB demonstrated a comparably high diagnostic accuracy as well in the elderly, as in the younger patient population, taking FFR as a reference method (AUC 0.906 vs. 0.866).

There are several limitations of our study. First, patient selection bias might influence study results, as individuals already scheduled for invasive coronary angiography were included in the original multicenter study. Likewise, the inclusion of patients from a predefined study group resulted in a skewed distribution of patients under 75 years old and above. Second, CMR stress perfusion was accomplished using a fixed standard dosage of adenosine adopted for patients’ weight, and no higher doses were administered. Thus, CMR might be false negative in some patients and MIB might be underestimated, especially as adequate stress response was not individually monitored (e.g., with the registration of patient symptoms or splenic switch-off). However, a mean change in heart rate ≥ 10 bpm from rest to stress (67 ± 11 vs. 84 ± 15) is a good marker of an adequate pharmacological stress reaction. Third, quantitative myocardial blood flow during stress CMR could not be measured, while MIB reflects the percentage of ischemia to myocardial volume in a selected time-point during first-pass myocardial perfusion. Fourth, CMR examinations were both performed at 1.5 T and 3.0 T and therefore the different signal-to-noise ratios might influence study results; however, a previous sub-study of the same trial did not find significant differences between the strength fields [21]. Fifth, FFR was considered significant and was not performed in lesions with a luminal narrowing on QCA > 80%, which might overestimate the CAD significance in the population; however, this corresponds to the standard technique. Lastly, the number of patients with advanced age in relation to the entire population was relatively small; 13% of the patients were aged ≥ 75 and 6% were ≥ 80 years old. Larger studies are needed with additional follow-up to determine the prognostic value of 3D CMR in this patient group.

In conclusion, myocardial ischemia testing is feasible in the elderly population with a high degree of accuracy using 3D CMR perfusion. Quantitative analysis of myocardial ischemia burden on 3D CMR perfusion is an excellent predictor of hemodynamically significant coronary lesions in patients with advanced age.

## Supplementary information

ESM 1 (PDF 168 kb)

## Abbreviations

CAD: Coronary artery disease
CMR: Cardiac magnetic resonance
ESC: European Society of Cardiology
FFR: Fractional flow reserve
LGE: Late gadolinium enhancement
LV: Left ventricle
MIB: Myocardial ischemic burden
QCA: Quantitative coronary angiography
SA: Short-axis
T: Tesla

## Declarations

### Guarantor

The scientific guarantor of this publication is Robert Manka.

### Statistics and biometry

One of the authors has significant statistical expertise.

### Study subjects or cohorts overlap

Some study subjects or cohorts have been previously reported in:

Manka R, Jahnke C, Kozerke S, et al Dynamic 3-dimensional stress cardiac magnetic resonance perfusion imaging: detection of coronary artery disease and volumetry of myocardial hypoenhancement before and after coronary stenting (2011). *J Am Coll Cardiol* 57:437–444.

Manka R, Paetsch I, Kozerke S, et al Whole-heart dynamic three-dimensional magnetic resonance perfusion imaging for the detection of coronary artery disease defined by fractional flow reserve: Determination of volumetric myocardial ischaemic burden and coronary lesion location (2012). *European Heart Journal* 33:2016–2024.

Manka R, Wissmann L, Gebker R, et al Multicenter evaluation of dynamic three-dimensional magnetic resonance myocardial perfusion imaging for the detection of coronary artery disease defined by fractional flow reserve (2015). *Circ Cardiovasc Imaging* 8(5).

Hamada S, Gotschy A, Wissmann L, et al Multi-centre study of whole-heart dynamic 3D cardiac magnetic resonance perfusion imaging for the detection of coronary artery disease defined by fractional flow reserve: gender based analysis of diagnostic performance (2017). *Eur Heart J Cardiovasc Imaging* 18:1099–1106.

### Methodology

• retrospective

• diagnostic or prognostic study

• multicenter study
