# Supplementary material for: Diagnostic performance of 3D cardiac magnetic resonance perfusion in elderly patients for the detection of coronary artery disease as compared to fractional flow reserve
Source: Eur Radiol. 2022 Aug 19;33(1):339–47. doi: 10.1007/s00330-022-09040-7 (PMC9755092; doi:10.1007/s00330-022-09040-7)
Supplement: Supplementary file 1 — (PDF 168 kb) [file 330_2022_9040_MOESM1_ESM.pdf]

Table 4. (Supplemental material) Diagnostic performance of 3D stress perfusion with CMR in octogenarians

	**< 80 years**<br>n = 390	**≥ 80 years**<br>n = 26	**p value**
**FFR**			
Sensitivity, % (95% CI)	87 (82-91)	89 (65-99)	0.853
Specificity, % (95% CI)	81 (74-86)	75 (35-97)	0.706
Positive predictive value, % (95% CI)	83 (79-87)	89 (70-96)	0.539
Negative predictive value, % (95% CI)	85 (79-89)	75 (43-92)	0.441

FFR: fractional flow reserve; CI: confidence interval.

Figure 3. (Supplemental material)

ROC curve of octogenarians aged ≥80 years for the prediction of pathological FFR using 3D stress perfusion CMR and MIB.

MIB: myocardial ischemic burden; ROC: Receiver operating characteristic; AUC: area under the curve.

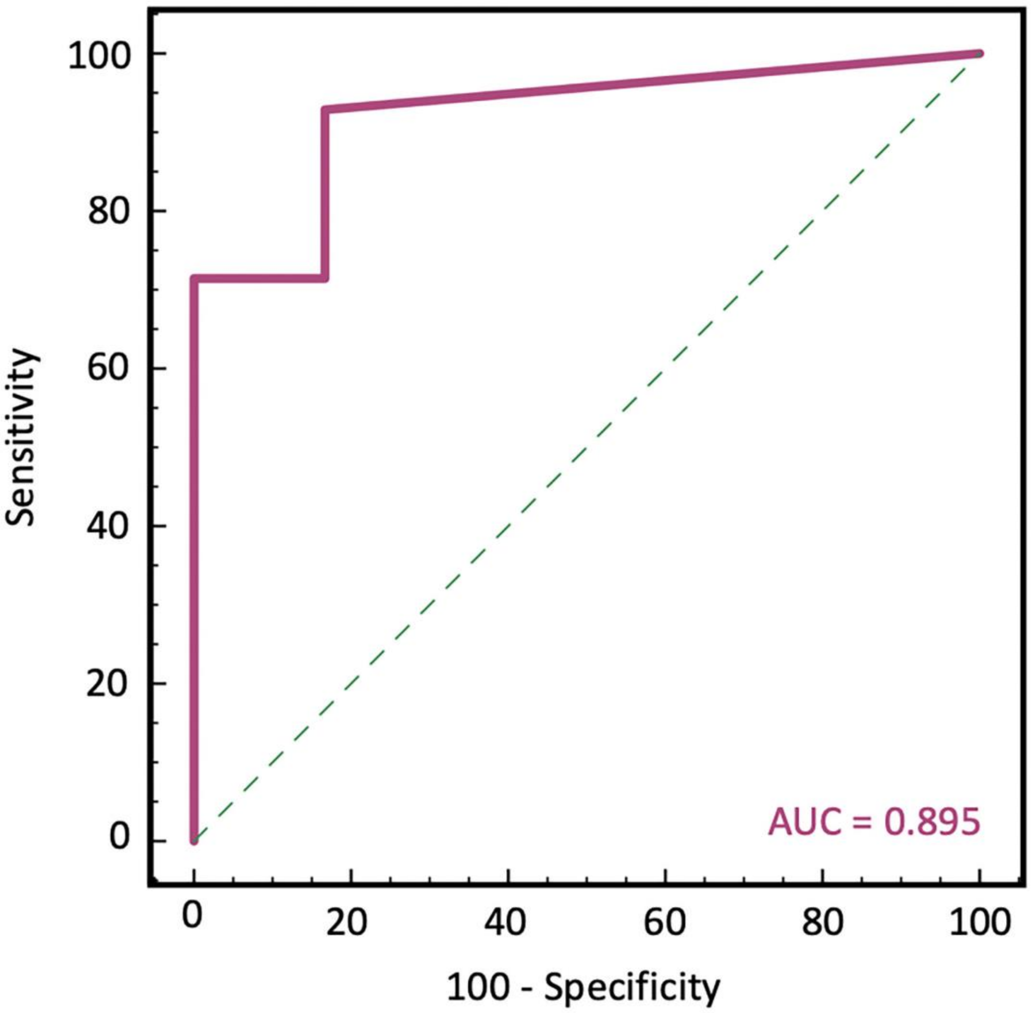
100
80
60
40
20
0
Sensitivity
AUC = 0.895
0
20
40
60
80
100
100 - Specificity
